# *In vivo* evaluation of an innovative synbiotics on stage IIIb-IV chronic kidney disease patients

**DOI:** 10.3389/fnut.2023.1215836

**Published:** 2023-06-15

**Authors:** Mirco Vacca, Giuseppe Celano, Francesco Maria Calabrese, Maria Teresa Rocchetti, Ilaria Iacobellis, Nadia Serale, Maria Calasso, Loreto Gesualdo, Maria De Angelis

**Affiliations:** ^1^Department of Soil Plant and Food Sciences, University of Bari, Bari, Italy; ^2^Department of Clinical and Experimental Medicine, University of Foggia, Foggia, Italy; ^3^Department of Precision and Regenerative Medicine and Ionian Area, University of Bari Aldo Moro, Bari, Italy

**Keywords:** chronic kidney disease, synbiotics, gut microbiota, gut metabolome, uremic toxins, indoxyl sulfate, p-cresyl sulfate

## Abstract

**Background:**

Microbiota unbalance has been proven to affect chronic kidney disease (CKD) patients and, noteworthy, microbiota composition and activity are implicated in CKD worsening. The progression of kidney failure implies an exceeding accumulation of waste compounds deriving from the nitrogenous metabolism in the intestinal milieu. Therefore, in the presence of an altered intestinal permeability, gut-derived uremic toxins, i.e., indoxyl sulfate (IS) and p-cresyl sulfate (PCS), can accumulate in the blood.

**Methods:**

In a scenario facing the nutritional management as adjuvant therapy, the present study assessed the effectiveness of an innovative synbiotics for its ability to modulate the patient gut microbiota and metabolome by setting a randomized, single-blind, placebo-controlled, pilot trial accounting for IIIb-IV stage CKD patients and healthy controls. Metataxonomic fecal microbiota and fecal volatilome were analyzed at the run-in, after 2 months of treatment, and after 1 month of wash out.

**Results:**

Significant changes in microbiota profile, as well as an increase of the saccharolytic metabolism, in feces were found for those CKD patients that were allocated in the synbiotics arm.

**Conclusions:**

Noteworthy, the here analyzed data emphasized a selective efficacy of the present synbiotics on a stage IIIb-IV CKD patients. Nonetheless, a further validation of this trial accounting for an increased patient number should be considered.

**Clinical trial registration:**

https://clinicaltrials.gov/, identifier NCT03815786.

## 1. Introduction

The entire human gastrointestinal (GI) tract is massively populated by microbes whose order of magnitude estimate is about 10^13^ cells per gram on the total dry weight content. This polymicrobial community is involved in the maintenance of the intestinal homeostasis and markedly contributes to human health (1, 2). An unbalanced gut microbiota negatively impacts on patients suffering from various GI diseases, including inflammatory bowel diseases (IBD), irritable bowel syndrome (IBS), and food-based diseases (e.g., lactose/fructose intolerance, celiac disease, or non-celiac gluten sensibility) (3–6). In addition to these mentioned GI-related pathologies, chronic kidney disease (CKD) has proven to affect GI microbiota composition and metabolic activity (7, 8).

Kidneys are responsible for most of the urea excreted from the body. Because of the kidney inability to remove metabolic waste products, CKD patients accumulated urea and its derivatives both in intestinal milieu and blood (9). In turn, the persisting GI accumulation of urea leads to the proliferation of microbial patterns encoding for genes involved in nitrogenous compound metabolism, therefore, exacerbating mechanisms dealing with uremic toxin increase. In this light, a field of research aimed at investigating the linkage between uremia status and gut microbiota (10–12). Together with the abnormal nitrogenous-accumulation, the signature of CKD-associated microbiota seems to be based on a decreased abundance in specific bacteria encoding for healthy saccharolytic pathways (e.g., *Bifidobacteriaceae*, *Lactobacillaceae*, *Lachnospiraceae*, *Ruminococcaceae*, and related sub-taxa) (13–16).

The manipulation of GI microbiota through probiotics-based therapies is an emerging strategy targeting different type of pathologies (17–19). In CKD patients, the probiotics administration is aimed at reducing the organic waste metabolites increased during uremic illness. In this pathologic condition, the administration of low-protein diets is not sufficient to completely avoid uremic retention solute accumulation (20, 21). However, probiotics consumption should be limited to prevent undesired effects. In fact, the growth of gut microbes encoding for ureases can increase the generation of ammonia and ammonium hydroxide that, in turns, leads to a reduced functionality of the intestinal epithelium (8). This condition increases the risk for lipopolysaccharides, proteins, or microbes present into the lumen to enter the blood stream (22). Hence, the concomitant presence of urea and urease enzymes in the colonic environment may lead to the complete loss of transepithelial electrical resistance and to the almost complete loss of the tight-junction functionality (23). Innovative formulations accounting for the combination of probiotics and prebiotics (i.e., synbiotics (24)) are one of the emerging strategies useful in modifying the microbiota high-proteolytic/low-saccharolytic metabolism ratio. Also, this strategy increases beneficial symbionts and reduces pathobiont relative abundances. Previous evidence investigated how very low protein diets (VLPD) supplemented with keto-analogues had an impact in terms of symbionts/pathobionts ratio (21, 25, 26). Due to its pivotal contribution in synthetizing protein-bound uremic toxins [i.e., indoxyl sulfate (IS) and p-cresyl sulfate (PCS)], the microbiota modulation has a key role in CKD worsening, and it is of great aid in decreasing the incidence of further associated comorbidities. Among these coexisting pathologies, the onset of cardiovascular events, inflammation, and endothelial dysfunction usually emerged (27).

In previous research, we described the useful workflow followed to combine probiotics, prebiotics, and antioxidants into an innovative synbiotics formulation (28). Thus, with a selective efficacy, it showed beneficial outcomes in clinical and physiological parameters by reducing free circulating IS and azotemia while improving the integrity of the small intestinal barrier (29). Furthermore, both abdominal pain and constipation syndromes are reduced with this treatment.

Based on these promising outcomes, the aim of the present study is to explore the effect of this innovative synbiotics on gut microbiota. To achieve this goal, the biological samples provided from both IIIb-IV nephropathy-stage CKD patients and healthy controls (HCs) during the single-blind, placebo-controlled, pilot trial previously described (29) were screened for metataxonomics and metabolomics analyses.

## 2. Materials and methods

### 2.1. Study design

The documentation for the pilot study, named NATURE 3.1, was filed to the Ethic Committee (EC). After ascertaining the compliance with the Declaration of Helsinki (IV Adaptation) dictates, in the EC session dated on the 22th of February 2017, the here used protocol was discussed and approved (n.5148 of 22.02.17). The EC positive opinion was acknowledged with a specific resolution of the General Director of the *Azienda Ospedaliero Universitaria Consorziale – Policlinico di Bari*, which decreed the authorization to conduct the pilot study named N.A.T.U.R.E. 3.1 (resolution No. 0643 of May 16, 2017). Closing the authorization procedures, the study was registered on ClinicalTrials.gov with the ID: NCT03815786.

#### 2.1.1. Definition of the treatment

As recently detailed (28), the innovative synbiotics NatuREN G® comprised a mixture of *Bifidobacterium animalis* BLC1 (10^9^ cells), *Lacticaseibacillus casei* LC4P1 (10^9^ cells), fructo-oligosaccharides (2.5 g), inulin (2.5 g), quercetin (640 mg), resveratrol (230 mg), and proanthocyanidins (13 mg). The placebo used in the present study was exclusively based on maltodextrins (500 mg) and aromas packaged in bags like those used to contain the innovative synbiotics.

#### 2.1.2. Enrollment and randomization of volunteers

**Figure 1:**
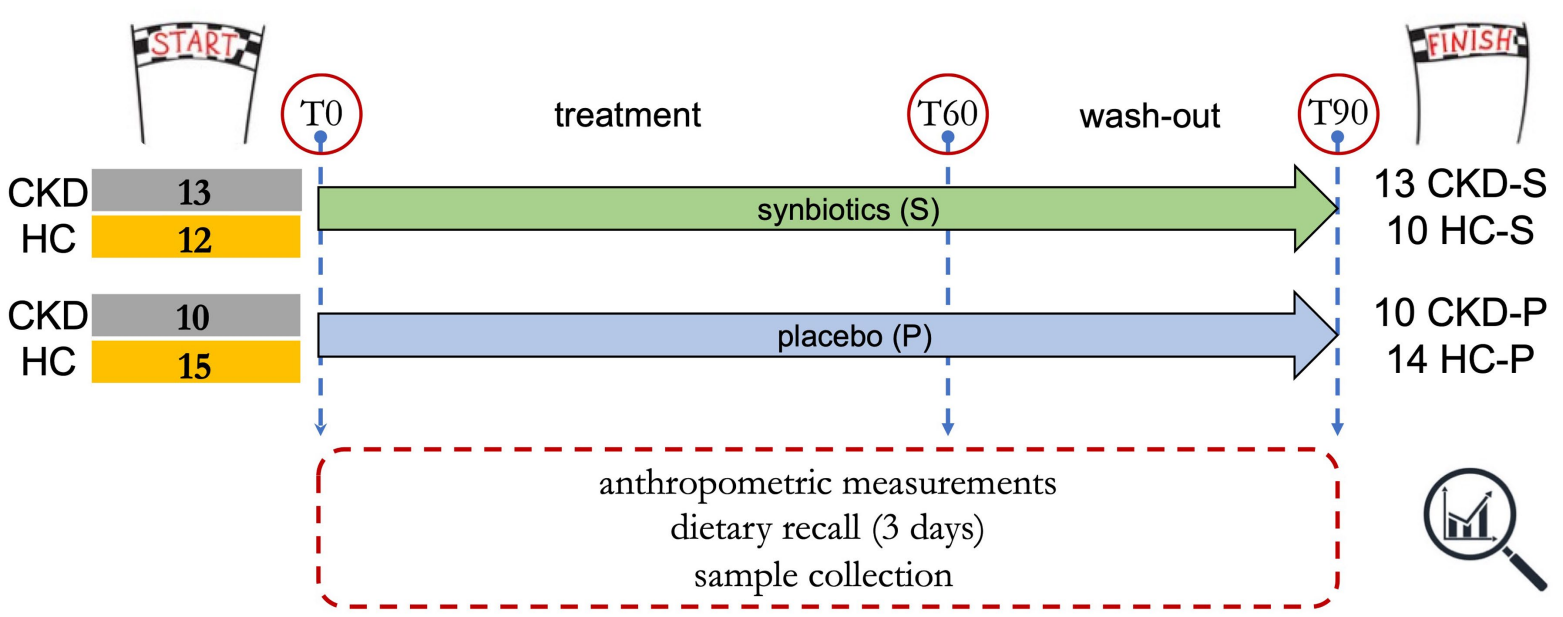
Study design.

The study design together with inclusion and exclusion criteria have been previously reported (29). All the enrolled participants signed an informed consent. The primary endpoint of the present research article consists in the detection of fecal microbiota changes once the 60 days of treatment have been completed. Briefly, the 50 enrolled subjects included 23 CKD patients (IIIb-IV stage) and 27 healthy controls (HCs) were randomly allocated in the synbiotics (S) or placebo (P) arm. Forty-seven out of 50 subjects completed the trial (Figure 1). Specifically, 13 CKD patients took the synbiotics (CKD-S) and 10 CKD patients belonged to the placebo arm (CKD-P). Ten HCs were treated with synbiotics (HC-S), and 14 HC took the placebo (HC-P). Stool samples were collected at the beginning of the study (T0), after 60 days of treatment (T60), and after additional 30 days of wash out (T90). At each time (T0, T60, and T90), a dietary recall of the last 3 days before the sample delivery was also collected. Compared to placebo, the synbiotics consumption would promote (i) a “new” intestinal microbiota balance, (ii) a decrease of bacterial taxa able to produce nitrogenous derivatives, and (iii) an increase in beneficial taxa and deriving metabolites.

### 2.2. Dietary recall elaboration

At each time point (T0, T60, and T90), a dietary recall considering the last 3 days was filled out by volunteers. To reduce bias, volunteers were instructed by the trained personnel of the research group on how to complete the template immediately after signing the informed consent. To volunteers, it was asked to list both foods and drinks taken in each one of the three-days taking care of marking the time at which the meal/snack was consumed as well as the weight or number of glasses (for foods and drinks, respectively). Moreover, volunteers were asked to include sauces and/or flavoring agents they consumed.

The dietary recalls were then analyzed by expert nutritionists by using the Winfood software [© 2022 WINFOOD SRL; Colonnella (TE), Italy] that returns the daily intake of nutrients, including minerals and vitamins.

### 2.3. Stool sample collection and total DNA extraction

At the collection, stool samples (~5 g/each) were immediately mixed with RNA later (Sigma-Aldrich, St. Louis, MO, United States) in a ratio 1:1 wt/vol, stored at −80°C, and subsequently used for total DNA extraction. An aliquot (500 μL) of each stool sample was used for total DNA extraction. Samples were diluted in 1 mL of PBS (phosphate buffer 0.01 M, pH 7.2) plus EDTA (0.01 M) solution and centrifugated (14,000 × g, 5 min, 4°C). This procedure was repeated twice to reduce the presence of PCR inhibitors before to proceed with the DNA extraction. The latter was carried out using the FastDNA® Pro Soil-Direct Kit (MP Biomedicals, CA, United States). The quality of the final DNA was checked by electrophoresis in agarose gel (1%) stained with Gel Red TM 10000X (Biotium, Inc.) and by spectrophotometric measurement at 260, 280 and 230 nm using the NanoDrop® ND-1000 Spectrophotometer (ThermoFisher Scientific Inc., MI., Italy).

### 2.4. Gut microbiota 16S rRNA NGS

Libraries for Next Generation Sequencing (NGS) were prepared starting from 12.5 ng of microbial rDNA, according to Illumina 16S Metagenomic Sequencing Library Preparation guide. The full-length of selected primers for amplify and sequencing the V1-V3 region of the 16S rRNA gene, including the Illumina overhang adapters, were Illumina 16S Amplicon PCR Forward Primer 27F (5′ – TCGTCGGCAGCGTCAGATGTGTATAAGAGACAGAGAGT TG ATCMTGGCTCAG) and Illumina 16S Amplicon PCR Reverse Primer 388R (5′ – GTCTCGTGGGCTCGGAGATGTGTATAA GAGACAGT GCTGCCTCCCGTAGGAGT). The enrichment of the target region was achieved through the following PCR amplification steps: preheating at 95°C for 3 min, 25 cycles of denaturation at 95°C for 30 s, annealing at 55°C for 30 s, extension at 72°C for 30 s, terminal extension at 72°C for 5 min. The amplified sequence was visualized using Bioanalyzer DNA 1000 chip (Agilent Technologies Inc., United States) to verify the size (about 500–600 bp) and the quality of the libraries. A second PCR amplification step was performed to attach Illumina dual indices and sequencing adapters. Finally, sequencing was performed on the Illumina MiSeq desktop sequencer, using paired 300 bp reads, and MiSeq v3 reagents (Illumina, United States). 16S sequencing-derived fastQ files were checked for quality using FastQC software (30). *In silico* bioinformatics analyses, including denoising and taxa assignment, were relied on the QIIME2 (31) microbiome platform (version 2020.8). QIIME plugin q2-deblur was used for the 16S denoising step. The SILVA 138 SSU database^1^ was used to infer the taxonomy starting from the ASV table.

Raw sequences as fastQ files are available within the NCBI BioProject repository (PRJNA872908)^2^.

### 2.5. qPCR in stool samples of CKD patients

Changes in *Lactobacillus* and *Bifidobacterium* genera were also verified by quantitative polymerase chain reaction (qPCR) and SYBR Green I chemistry as previously described (32). Primer details are summarized in Supplementary Table S1. The genomic DNA was extracted by Bacterial Genomic DNA Isolation Kit (Norgen Biotek Corp., Canada) and quantified by spectrophotometric measurement using the NanoDrop® 2000c Spectrophotometer (ThermoFisher Scientific Inc., MI., Italy). qPCR reactions were made following a holding stage at 95°C for 20 s. Then a cycling stage made at 95°C for 5 s, plus an annealing step of 30 s (Supplementary Table S1), and an extension of 35 s were repeated for 40 times, followed by a last step run at 94°C for 15 s. The qPCR amplicon melting curve analysis started at a temperature of 60°C and increased of 1°C/5 s until the final temperature of 95°C. Quantifications were made by a RotorGene 6,000 (Corbett Research Ltd., Australia) and the RotorGene Q Series Software 2.3.1 Release (QIAGEN, Hilden, Germany). Reactions were prepared with 1 μL (40 ng of template), 2× SsoAdvanced™ Universal SYBR® Green Supermix (Bio-Rad Laboratories S.r.l., Milano, Italy) and 0.2 nM of each primer (Life Technologies Italia, Italy). All results were expressed as the average of the cycle thresholds (Ct) from two independent experiments.

### 2.6. Fecal metabolome analysis

Stool samples (2 g) were added with 10 μL of internal standard solution (4-methyl-2-pentanol) at 33 ppm, placed into 20 mL glass vials, and sealed with polytetrafluoroethylene (PTFE)-coated silicone rubber septa (20 mm diameter) (Supelco, Bellefonte, PA, United States). To obtain the best extraction efficiency, the micro-extraction procedure was performed as previously described (33), with slight modifications. A conditioned 50/30 μm DVB/CAR/PDMS fibre (Supelco, Bellefonte, PA, United States) was used to obtain VOC adsorption. The Clarus 680 (Perkien Elmer) gas-chromatography was equipped with a capillary column Rtx-WAX (30 m × 0.25 mm i.d., 0.25 μm film thickness) (Restek, Bellfonte, PA, United States) and a single quadrupole mass spectrometer Clarus SQ 8C (Perkien Elmer) was coupled with the gas chromatography system. The generated GC–MS chromatograms were singularly analyzed for peak identification using the National Institute of Standard and Technology 2008 (NIST) library. A peak area threshold (> 1,000,000 and 85% or greater probability of match) was used for VOC identification followed by visual inspection of the fragment patterns. Quantitative data for the identified compounds were obtained by interpolating the relative areas versus the internal standard area.

Mass spectrometry data were deposited at EMBL-EBI MetaboLights database under the personal identifier MTBLS5804.

### 2.7. Indoxyl sulfate and p-cresol sulfate quantification

Circulating levels of IS and pCS were determined as previously detailed (29). To determine total and free IS and PCS in plasma samples, a Multiple Reaction Monitoring (MRM) analysis was carried out using a triple quadrupole mass spectrometer (API4000, AB SCIEX, Carlsbad, CA, United States) equipped with an ESI source and online connected to high-performance liquid chromatography (HPLC) system (CBM-20A LC, Shimadzu, Kyoto, Japan). All the samples were run in duplicate to reach an intra-assay coefficient of variation <10%.

### 2.8. Statistical analyses

As appropriate, variables were reported as mean ± standard deviation (SD), median and interquartile range (IQR), or count (percentage). The microbiota composition was analyzed at different taxonomic levels with the MaAsLin2 R package (huttenhower.sph.harvard.edu/maaslin/) based on metadata stratification (subgrouping). For MaAsLin2 regression model, only those features (ASV relative abundance) showing a concomitant value of *p* (*P*) <0.05 and q-value (qval) <0.5 were considered as statistically significant differences. The software Statistica version 7.0 (StatSoft, Vigonza, Italia), GraphPad Prism version 8.4.0 (GraphPad Software, San Diego, CA, United States), and MetaboAnalyst version 5.0 (metaboanalyst.ca/; accessed online 6 July 2022) were used to elaborate and visualize data. The latter software was also used to correlate ASV relative abundance and uremic toxin concentrations according with Spearman’s rank regression model and setting R^2^ > |0.5| and *p* < 0.05 as the significance threshold.

## 3. Results

### 3.1. Dietary intake assessment

The dietary recalls of the last 3 days before sample collection were used to evaluate the symbiotics treatment contribution by verifying whether randomization, in terms of the volunteer’s dietary habits, acted as a confounding factor.

At the run-in (T0), CKD-P showed a lower intake of energy, sodium, potassium, and folic acid with respect to CKD-S (Supplementary Table S2). However, except for sodium, differences in energy, potassium, and folic acid values were not found at the subsequent follow-ups.

In HCs profiled at T0, those volunteers allocated in HC-S arm had a lower intake of potassium and vitamin A than HC-P. At T60, only the potassium was one more time lower in HC-S than HC-P.

Concerning the temporal changes within the same arm, CKD-S and HC-S reported a higher fiber intake at T60 than T0. In line with this, lower values of the soluble fiber fraction and proteins/fibers ratio were found at T60. The absence of these differences at T90 suggested a direct contribute of the synbiotics excluding the hypothesis of changes in volunteers’ dietary habits.

### 3.2. Illumina MiSeq data analyses

Total bacteria in volunteer stool samples were analyzed by 16S metataxonomic sequencing. The GI microbiota alpha diversity (Shannon’s index) was reported in the Supplementary Table S3. Aiming at verifying an optimal randomization process, no differences existed in ASV number and Shannon’s index at the run-in (T0) for both CKD patients and HCs allocated in P and S arms.

After 60 days (T60), the synbiotics treatment led to an increased number of ASVs in both CKD patients and HCs. Noteworthy, this effect was also found at the end of the washout (T90) only in CKD-S.

The only noticed Shannon’s index difference emerged in HC-P-T60, which showed higher values than themselves at T0 and HC-S-T60.

Before performing subsequent comparisons, the sample randomization was verified by evaluating the variable contribution in a principal component analysis (PCA). Looking at PCA sample closeness, neither CKD nor HC cohorts (Supplementary Figures S1A,B) appeared as distinct clouds based on P and S stratification. The inspection of 16S metataxonomic abundances revealed how Firmicutes, Bacteroidetes, Proteobacteria, and Actinobacteria described more than 98% of total sequences in all samples. Phyla with a relative abundance lower than 0.1% were merged and indicated as “Other” (Supplementary Figure S2).

### 3.3. Temporal changes of gut microbiota

According to MaAsLin2 regression model (Supplementary Table S4), metataxonomic differences within each subgroup (CKD-P, CKD-S, HC-P, and HC-S) were investigated comparing the follow-ups (T60 and T90) against the related baseline (T0). Based on *p* < 0.05 and FDR, only features (ASVs) showing qval <0.5 were considered as statistically significant differences.

In CKD-S, the synbiotics led to a significant shift in the Firmicutes/Bacteroidetes ratio. In fact, Firmicutes was found to be positively modulated by the innovative synbiotics treatment at both follow-ups, whereas Bacteroidetes had an opposite behavior. At family level, *Coriobacteriaceae* and *Flavobacteriaceae* were positively and negatively affected by the synbiotics, respectively, also exhibiting a carry-over effect till the end of the trial (T90). *Blautia* was the only genus positively modulated by the synbiotics. Other taxa, indeed, showed statistically significant variations in terms of qval <0.5; however, none of them displayed the qval lower than the significance threshold for both the follow-ups (T60 and T90).

No statistically significant differences involved the CKD-P arm.

As far as it concerns the HCs, the administration of the synbiotics treatment revealed its weak capacity in modulating the gut microbiota based on the absence of significant differences between T60 and T90 against T0.

In HC-P, except for *Lachnospira*, no additional significant differences were found in both the follow-ups mimicking occasional microbial fluctuations during the trial.

### 3.4. Metataxonomic differences between arms after 60 days of trial

**Figure 2:**
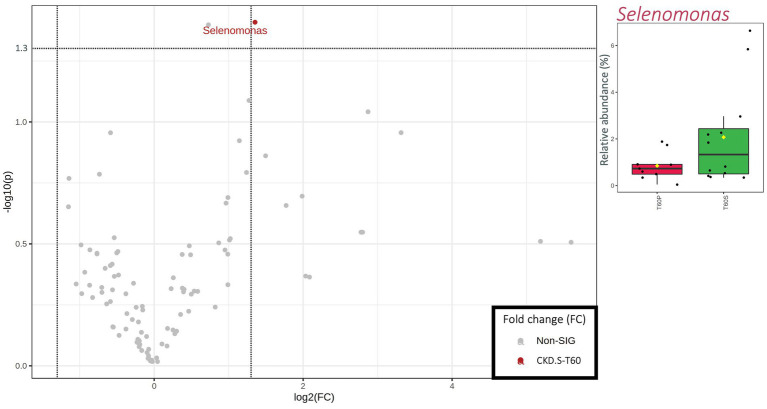
Scatterplot (Volcano plot) and boxplots (red for CKD-P and green for CKD-S) showing the metataxonomic differences in gut microbiota of chronic kidney disease (CKD) patients based on 60 days of treatment (T60) with placebo (P) or synbiotics (S). Significance was reached for taxa showing both a fold change (FC) >1.35 log_2_ and a value of *p* (−log_10_) >1.30. In boxplots, statistically significant taxa emerged from a nonparametric Wilcoxon rank-sum test combined with fold change (FC) analysis.

Differences between S and P arms in terms of metataxonomics relative abundances were restricted to 60 days of treatment. Comparing CKD-S to CKD-P, only *Selenomonas* was significantly different in patients since this taxon showed a higher relative abundance in the former arm of patients (IQR = 0.50–2.44%) than the latter arm (IQR = 0.49–0.90%) (Figure 2).

**Figure 3:**
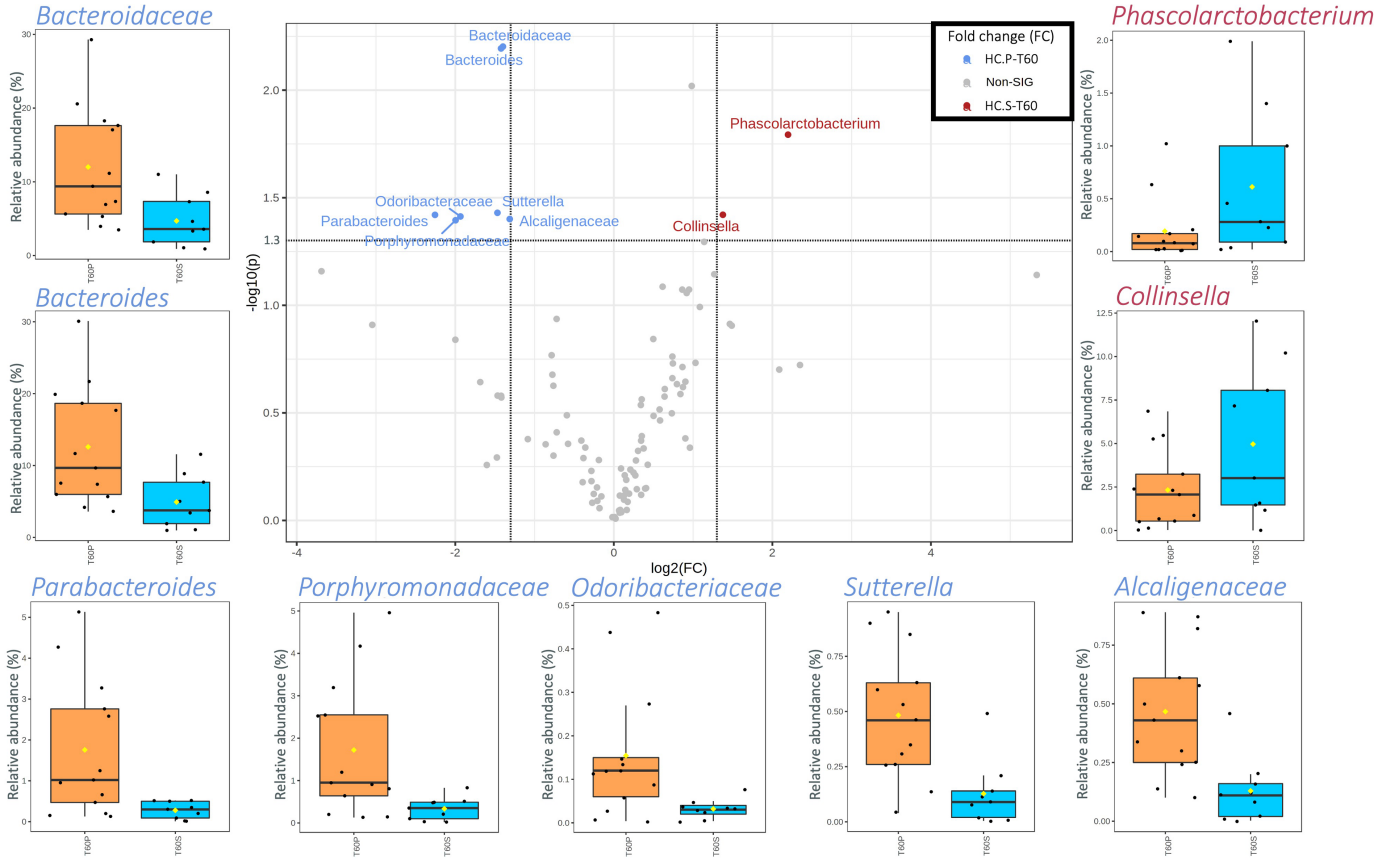
Scatterplot (Volcano plot) and boxplots (orange for HC-P and light blue for HC-S) showing the metataxonomic differences in gut microbiota of healthy controls (HC) based on 60 days of treatment (T60) with placebo (P) or symbiotic treatment (S). Significance was reached for taxa showing both a fold change (FC) log_2_ > 1.35 and a value of *p* (−log_10_) >1.30. In boxplots, statistically significant taxa emerged from a nonparametric Wilcoxon rank-sum test combined with fold change (FC) analysis.

A higher number of taxa was significantly different between HC-P and HC-S after 60 days of treatment (Figure 3). HC-P mainly harbored various families and genera belonging to Bacteroidetes phylum (i.e., *Bacteroidaceae*, *Odoribacteraceae*, *Porphyromonadaceae*, *Bacteroides*, and *Parabacteroides*). In addition, two taxa belonging to β-Proteobacteria (i.e., *Alcaligenaceae* and *Sutterella*) were higher in HC-P than HC-S. Instead, *Phascolarctobacterium* and *Collinsella* had a higher abundance in HC-S than HC-P samples.

### 3.5. The synbiotics affects gut microbiota profiles in CKD patients

The MaAsLin2 regression model enlightened how the synbiotics administration mainly shaped the gut microbiota of CKD patients. Being confident of this result, we downstream investigated the overall profile of the gut microbiota in this subgroup during the trial (taxa with a relative abundance >0.1% within all samples and prevalence >20%).

**Figure 4:**
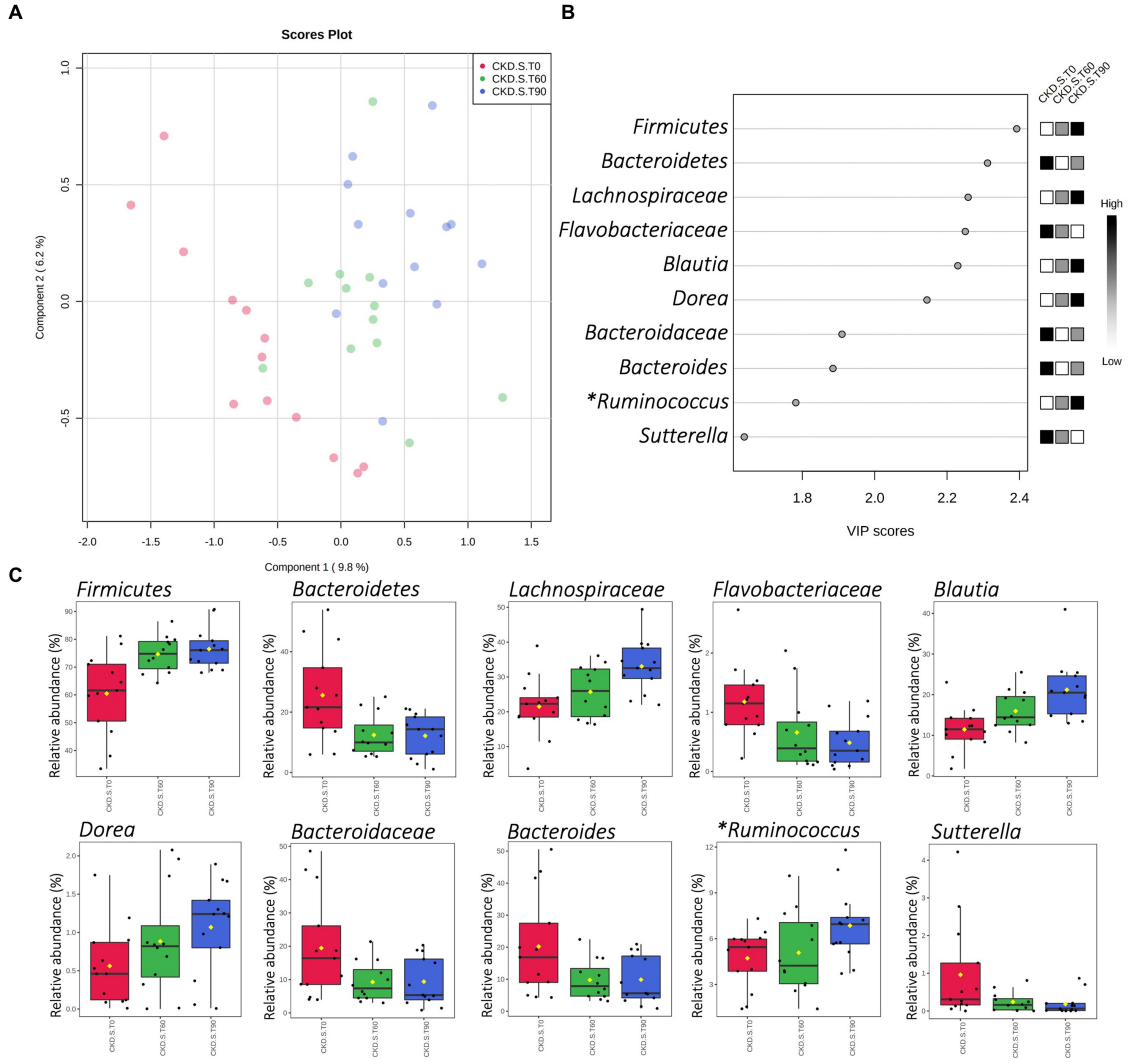
Partial Least-Squares Discriminant Analysis (PLS-DA) of gut microbiota in CKD patients treated with symbiotic (CKD-S). Score plot and VIP scores **(A,B)** of samples delivered at run-in (T0), after 60 days of treatment (T60), and after 30 days of wash-out (T90). **(C)** Shows the CKD-S relative abundances (16S rRNA NGS) of those bacterial taxa that were included in the top 10 of VIP scores. (*) Genus *Ruminococcus* hierarchically belonging to *Ruminococcaceae* family.

A partial least-squares discriminant analysis (PLS-DA) was applied with the aim of verifying those taxa that mainly described the differences occurred in CKD-S after treatment (T60) and wash out (T90). As evidenced by PLS-DA, the CKD-S cloud at T0 was placed apart in terms of linear distance from the ones relative to the T60 and T90. Furthermore, according to the first component, the distance between T0 and T90 clouds was greater than the one existing between T0 and T60 ones, thus suggesting a carry-over effect (Figure 4A). Based on the “scores of variable importance on projection” (VIP scores), the top 5 variables (i.e., Firmicutes, Bacteroidetes, and *Lachnospiraceae*, *Flavobacteriaceae*, and *Blautia*) confirmed the result found by applying the MaAsLin2 regression model. Other additional relevant variables, ranked in the top 10 list, included *Dorea*, *Bacteroidaceae*, *Bacteroides*, *Ruminococcus* (belonging to the family of *Ruminococcaceae*), and *Sutterella* (Figures 4B,C).

### 3.6. qPCR In CKD patients

**Figure 5:**
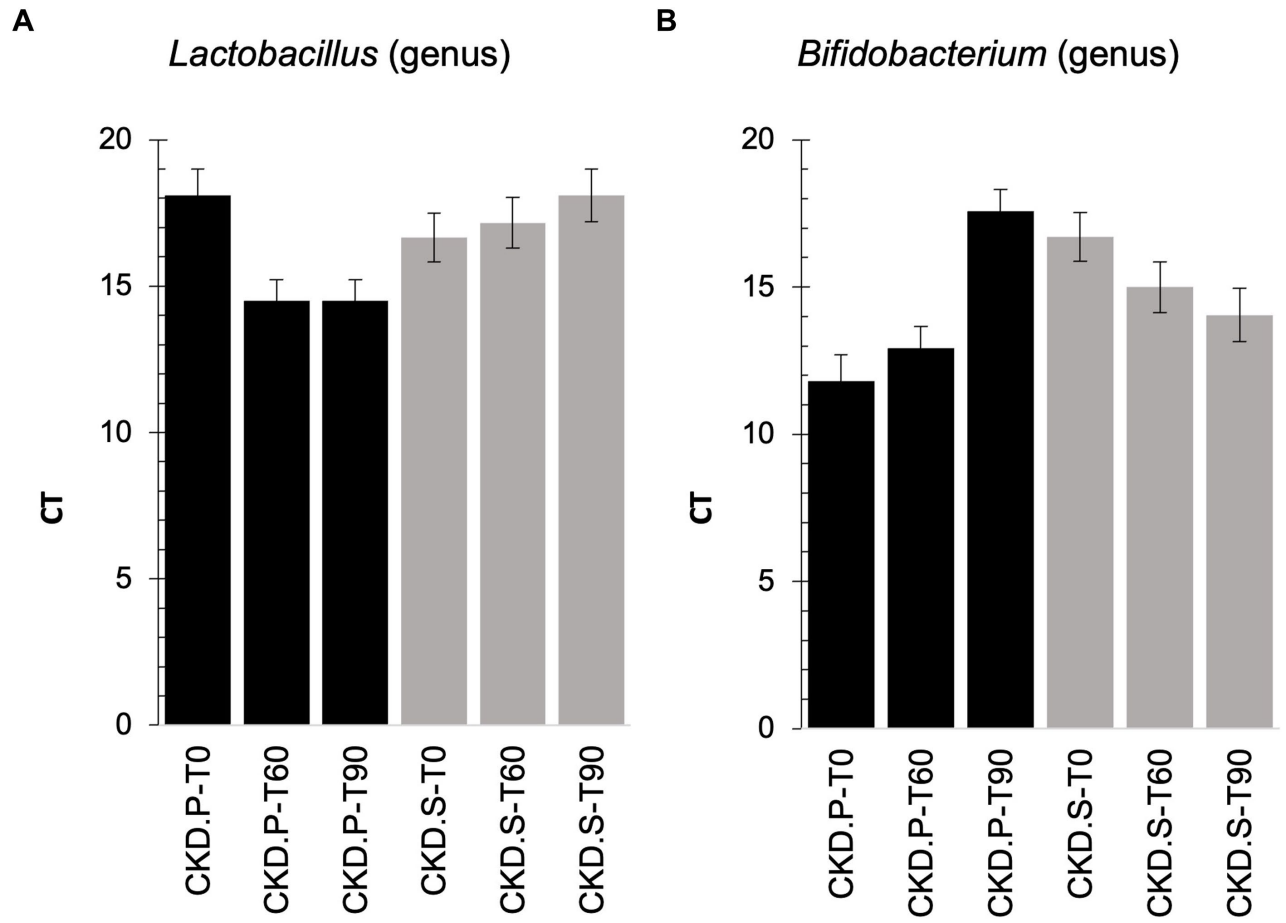
qPCR on *Lactobacillus* **(A)** and *Bifidobacterium* **(B)** genera found in feces of chronic kidney disease (CKD) patients at baseline (T0), treated for 60 days (T60) with the synbiotics (S) or treated with the placebo (P), and after the wash out (T90).

Due to the lack of metataxonomic results concerning the administered probiotics, a qPCR analysis was carried out to deeply explore this field in CKD patients, and it revealed how the cycle threshold (CT) of the *Lactobacillus* genus was higher in CKD-P than CKD-S at the run-in (Figure 5). After following 60 days of treatment, the qPCR analysis showed an increase of *Lactobacillus* CT-value in CKD-S. A further increase of *Lactobacillus* CT-value was assessed in CKD-S at T90, while no difference concerned the group of CKD-P. An opposite trend was found for *Bifidobacterium* that exhibited a higher CT value in CKD-S than CKD-P. By singularly inspect the two groups using the treatment time as the variable for sample stratification, *Bifidobacterium* CT-value decreased in CKD-S whereas increased in CKD-P.

### 3.7. Correlations between uremic toxins and gut microbiota

**Figure 6:**
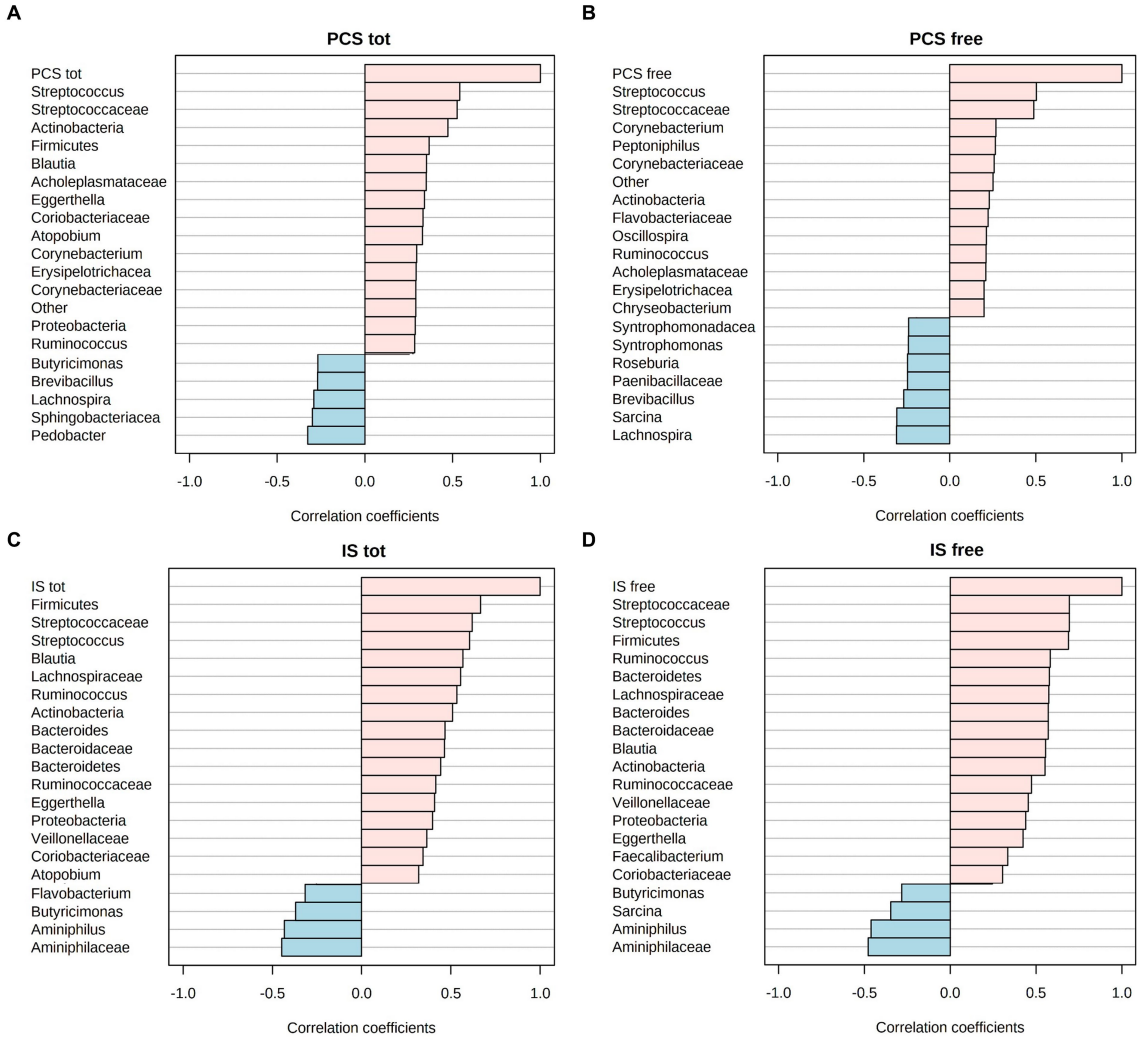
Spearman R-correlation coefficient (R^2^) between uremic toxin concentrations, i.e., free and total p-cresyl sulfate (PCS) **(A,B)** or free and total indoxyl sulfate (IS) **(C,D)**, and the corresponding top-20 gut microbiota taxa. Positive values of correlation are flagged with pink color, while negative values in light blue.

Microbiota ASV relative abundances were correlated with the concentration of both free and total IS and PCS. The investigation was carried out considering all sampling times (from T0 to T90) in all CKD patients, treated both with the placebo and the synbiotics formulation. The Spearman’s rank correlation analysis showed that *Streptococcaceae* (and *Streptococcus*) positively correlated with both forms (free and total) of IS and PCS (Figure 6). Interestingly, the above-mentioned correlation coefficients were higher than 0.5 in all correlation bar-plots.

### 3.8. Fecal metabolome

Fecal volatile organic compounds (VOCs) were investigated in both CKD and HC arms. The profiling was carried out at T0 and after 60 days of intervention (T60). A total of 175 VOC-peaks have been identified in stools (data not shown). Based on the chemical belonging class, VOCs were grouped in alcohols (23), aldehydes (18), aromatic heterocyclic (16), esters and methyl esters (34), hydrocarbons (21), ketones (13), phenols (3), lactones (1), short and medium chain fatty acids (SCFA and MCFA, respectively) (11), sulfur compounds (3), terpenes (23), and others (5). The metabolic profiles broadly varied between groups after treatment. However, some significant differences need to be reported (Supplementary Table S5). After randomization (T0), 2-undecanone, hexadecanol, and octanal compounds were significantly different in CKD (S vs P), while durene in HC. At T60, the comparison of S vs P indicated that 3-carene and o-cymene were significantly different. In detail, compared to the relative P-groups, 3-carene was more abundant in CKD-S (*p* = 0.009) while o-cymene (*p* = 0.027) was less abundant in HC-S.

Acetic and propanoic acids (*p* ≤ 0.048) increased in CKD-S compared to the relative baseline (T0). Also, 2-tridecanone and decane significantly increased (*p* ≤ 0.048), whereas dimethyl trisulfide and nonanoic acid showed the opposite trend (*p* ≤ 0.044). In CKD-P, an increase of carbon disulfide, 2-carbomethoxyphenol, γ-terpinene, and 3-ethyltoluene occurred at T60 (*p* ≤ 0.035). In HC-S at T60, a significant decrease of carboxylic acids (iso-butyric acid and 3-methylvaleric acid) and their derivative esters (ethyl butyrate, butyl butyrate, butyl valerate, ethyl valerate, propyl valerate, ethyl 4-methylpentanoate, and ethyl pentadecanoate) was found. Similarly, 1-pentanol, 2-nonedecanone and estragole showed a significant decrease. Instead, at the same time of sampling, HC-P showed an increase of 2-undecanone, 2-tridecanone and heneicosane (*p* ≤ 0.036), together with a decrease of ethyl phenylacetate (*p* = 0.032).

## 4. Discussion

The present study explored the effectiveness of an innovative synbiotics treatment useful in modulating the gut microbiota of CKD patients compared with HC volunteers. In a previously published study, the same formulation proved to reduce free circulating IS and azotemia in CKD patients while not in HC (29) and to counteract both abdominal pain and constipation syndromes. Furthermore, this formulation improved the small intestine barrier integrity in CKD patients (29), a condition that, especially in case of comorbidity presence, has been previously associated with a high abundance of Proteobacteria exacerbating the related pathological traits (35). Various taxa belonging to the Proteobacteria phylum, indeed, are linked to metabolic pathways leading to the epithelial disruption and, consequently, facilitating gut colonization by exogenous pathogens (36, 37). A scenario that was confirmed by previous evidence enlightening how some Proteobacteria species entered the blood stream in end-stage renal disease (ESRD) patients (38). Proteobacteria can encode for urease, uricase, p-cresyl-and indole-forming enzymes (13). Therefore, being potential catalysts for uremic toxin metabolism, Proteobacteria are also associated to CKD worsening (13, 27, 34, 39). However, this role is not exclusively exerted by Proteobacteria. An *in-silico* study noticed that even some bacterial families belonging to Firmicutes (e.g., *Bacillaceae*, *Clostridiaceae*), Bacteroidetes (e.g., *Bacteroidaceae*, *Flavobacteriaceae*, *Prevotellaceae*), and Verrucomicrobia have genes that can be considered as “unhealthy” for patients affected by CKD (13). In the present study, although the synbiotics formulation did not affect the Proteobacteria abundance, it modulated the gut community of CKD patients by increasing Firmicutes/Bacteroidetes ratio. A decrease in this ratio was previously considered to be a signature of chronic relapsing inflammation affecting the intestinal mucosa (40). Although species belonging to Firmicutes and Actinobacteria contain relatively few fiber-metabolizing enzymes per organism, these phyla are the main responders to plant-derived nutrients (41). Both Firmicutes and Actinobacteria exert specialized roles, including the initiation of complex substrate degradation (42). Therefore, the increased intake of fiber (assessed by dietary recall after the treatment) may sustain the abundance of Firmicutes, as mainly linked to the *Lachnospiraceae* increasing tendency. *Lachnospiraceae* is one of the core gut bacterial families known for its marked saccharolytic metabolism. Many species belonging to this taxon can synthesize SCFA (43), which are postbiotics used as the major energy source by bowel epithelial cells (44). It is worth mentioning that increased levels of *Lachnospiraceae* have been previously assessed both in humans and animals with CKD (7, 8). This reflects the controversial role of this bacterial family (45). In fact, the metabolic contribute of *Lachnospiraceae* to healthy needs to be carefully considered based on changes in physio-pathological parameters. With a specific respect to our CKD patient cohort, Cosola and colleagues (29) previously assessed an improvement of the small intestinal barrier integrity, a condition suggesting a beneficial effect on the whole metabolism supported by *Lachnospiraceae* abundances. A similar result was reported by Rossi et al. (46), who set a 6 weeks-long synbiotics-based trial with CKD patients and observed a decreased concentration of serum PCS with a simultaneous increase in *Lachnospiraceae* abundances. The synbiotics used by Rossi et al. (46) did not contain antioxidant compounds. Nonetheless, studies in pigs and rats showed how *Lachnospiraceae* were positively modulated by a nutritional supplementation enriched in polyphenols (47, 48).

In our analyses, both MaAsLin2 regression model and PLS-DA showed that *Flavobacteriaceae* (Bacteroidetes phylum) were negatively affected by the innovative synbiotics administration. This family is mainly associated with the aquatic microbiota (49). However, some *Flavobacteriaceae* sub-taxa have been shown to colonize the human gut microbiota and are known to be opportunistic pathogens in hosts with a compromised immune system (50).

A relevant contribution in decreasing the abundance of Bacteroidetes at phylum level was mainly determined by *Bacteroidaceae* and more in-depth by *Bacteroides* genus, as resulted from our PLS-DA. Previously, *Bacteroides* were positively correlated with dietary regimens enriched in fats and animal proteins (21, 51). For this reason, the concomitant presence of highly abundant *Bacteroides* percentage and a higher protein dietary intake may increase the possibility to detect potential detrimental metabolites, as derived from the fermentation of amino acids (52). An increase in these sub-metabolites (e.g., branched-chain fatty acids, ammonia, amines, p-Cresol, sulfides, indole-compounds, or hydrogen sulfide) alters the filtration capacity leading to their over-accumulation in the intestinal milieu and blood stream of CKD patients (9). The shift from a mainly proteolytic to a high saccharolytic fermentation is likely to inhibit the protein fermentation, instead. This would counteract many of the detrimental effects that have been associated to unbalanced diets (53). The high proteolytic fermentation, at the colon level, positively correlates with high concentrations of uremic toxins, mostly accounting for IS and PCS. Due to the difficulty in removing these waste products, nutritional managements aim to prevent their synthesis. Herein, we found that *Streptococcaceae* (in particular, *Streptococcus*) correlated with both IS and PCS, thus suggesting the positive involvement of this taxa in their synthesis. Literature reports a positive contribution given by *Streptococcus* in reducing uremic toxins (54). Contrarily, Yang and colleagues (55) found a higher abundance of *Streptococcus* in nephropathic patients under hemodialysis. Similarly, in a previous trial, we also assessed that a VLPD regimen significantly reduced the abundance of *Streptococcaeae* participating to proteolytic fermentations even at gut level (56).

To address how the synbiotics administration may affect gut microbiota metabolism, we inspected the fecal VOC profiles and observed that its administration induced a significant increase of acetic and propionic acids in CKD. The acetic acid metabolism is greatly affected by the balance between saccharolytic and proteolytic fermentation and by the ingestion of acetogenic fibers (57). Evidence also suggests a possible contribution of a fasting-induced acetic acid synthesis as derived by a cross-feeding mechanism based on an increased Firmicutes/Bacteroidetes ratio (58). This is in line with the significant microbial differences that we found here. The pathways for the acetic acid synthesis are largely present among GI bacterial phylogenetic groups. Differently, pathways involved in propionic acid production seem to be highly conserved in specific taxa and modulated by specific substrates (59). Propionic acid is mainly produced through succinate and propanediol pathways, as the result of the carbohydrates metabolism (60). The succinate pathway was previously assessed in Bacteroidetes and *Negativicutes* (class of Firmicutes) genomes (61), while the propanediol pathway was observed in gut commensal bacteria belonging to *Lachnospiraceae* (in particular, *Roseburia inulinivorans* and *Blautia*) (62, 63). Hence, we can advisedly suggest how the increase of propionic acid found in CKD patients who underwent the synbiotics treatment well fits with the significant increase of *Lachnospiraceae*. Looking at the proteolytic metabolism, the dimethyl trisulfide decrease in CKD-S allowed us in speculating on the reduction of sulfur-containing substrates. Compared to healthy animals under the same dietary regimen, Meinardi and colleagues (64) found a higher concentration of dimethyl-disulfide and trisulfide in fecal samples from CKD rats. This outcome can be also analyzed in combination with the increase of sulfur compounds (e.g., carbon disulfide) found in CKD-P. In fact, the concentration of carbon disulfide is positively affected by GI bacterial detoxification mechanisms (65). On the other hand, the effectiveness of the treatment in HC samples mainly accounts for a decrease in relative concentrations of esters. Moreover, no outcome was significantly related to both SCFA synthesis and proteolytic metabolism.

## 5. Conclusion

Our efforts were focused on the possibility of reducing CKD-related dysbiosis and limiting the CKD progression through dietary interventions or probiotic administration, prebiotics, or both. We investigated the effectiveness of a synbiotics formulation targeting CKD, and we verified its selective efficacy on stage IIIb-IV CKD patients. The treatment was able to modulate the gut microbiota and the related metabolisms while, as previously shown (29), it also exerted some beneficial effects resulting by the inspection of specifically related clinical parameters (i.e., reducing free circulating IS, improving small intestine barrier integrity, and ameliorating both abdominal pain and constipation syndromes). In terms of annotated taxa abundances, the formulation administration increased the Firmicutes/Bacteroidetes ratio as also reflected by the increase in the saccharolytic metabolism and the concomitant reduction of the proteolytic-one. Therefore, the present work paves the way toward the setting of further studies on nutritional managements and adjuvant therapies based on probiotics and prebiotics administration in diseases without an aetiology strictly associated to the GI tract, as in the case of nephropathies. Furthermore, to better profiling the new gut microbiota balancing, a higher number of patients is required to permit new stratifications of volunteers, based on phenotype-impacting clinical parameters like glomerular filtration rates and dietary habits.

## Data availability statement

The datasets presented in this study can be found in online repositories. The names of the repository/repositories and accession number(s) can be found in the article/Supplementary material.

## Ethics statement

The studies involving human participants were reviewed and approved by the Azienda Ospedaliero Universitaria Consorziale - Policlinico di Bari. The patients/participants provided their written informed consent to participate in this study.

## Author contributions

MV, GC, MR, and MC: methodology. MV, GC, MR, II, and NS: investigation. MV, GC, and FC: data elaboration and statistical analyses. MV, FC, and GC: writing–original draft preparation. MV, GC, and FC: writing–review and editing. LG and MA: conceptualization, validation, supervision, and project administration. All authors contributed to the article and approved the submitted version.

## Funding

This work was funded from the XUANRO4 - NATURE 3.1 - *Nuovo Approccio Per la Riduzione Delle Tossine Uremiche Renali*, REGIONE PUGLIA - FSC 2007–2013 Ricerca. Intervento “*Cluster Tecnologici Regionali*.”

## Conflict of interest

The authors declare that the research was conducted in the absence of any commercial or financial relationships that could be construed as a potential conflict of interest.

## Publisher’s note

All claims expressed in this article are solely those of the authors and do not necessarily represent those of their affiliated organizations, or those of the publisher, the editors and the reviewers. Any product that may be evaluated in this article, or claim that may be made by its manufacturer, is not guaranteed or endorsed by the publisher.

## Footnotes

^1^ https://www.arb-silva.de/documentation/release-138/

^2^ http://www.ncbi.nlm.nih.gov/bioproject/872908
