# Supplementary material for: In vivo evaluation of an innovative synbiotics on stage IIIb-IV chronic kidney disease patients
Source: Front Nutr. 2023 Jun 15;10:1215836. doi: 10.3389/fnut.2023.1215836 (PMC10311028; doi:10.3389/fnut.2023.1215836)
Supplement: Supplementary file 1 [file Data_Sheet_1.docx]

Supplementary Material

In vivo evaluation of an innovative synbiotics on stage IIIb-IV chronic kidney disease patients

Mirco Vacca ^1,†^, Giuseppe Celano ^1,†^, Francesco Maria Calabrese ^1,*^, Maria Teresa Rocchetti ^2^, Ilaria Iacobellis ^1^, Nadia Serale ^1^, Maria Calasso ^1^, Loreto Gesualdo ^3^ and Maria De Angelis ^1^

*** Correspondence:** Francesco Maria Calabrese, francesco.calabrese@uniba.it

# Supplementary Figures


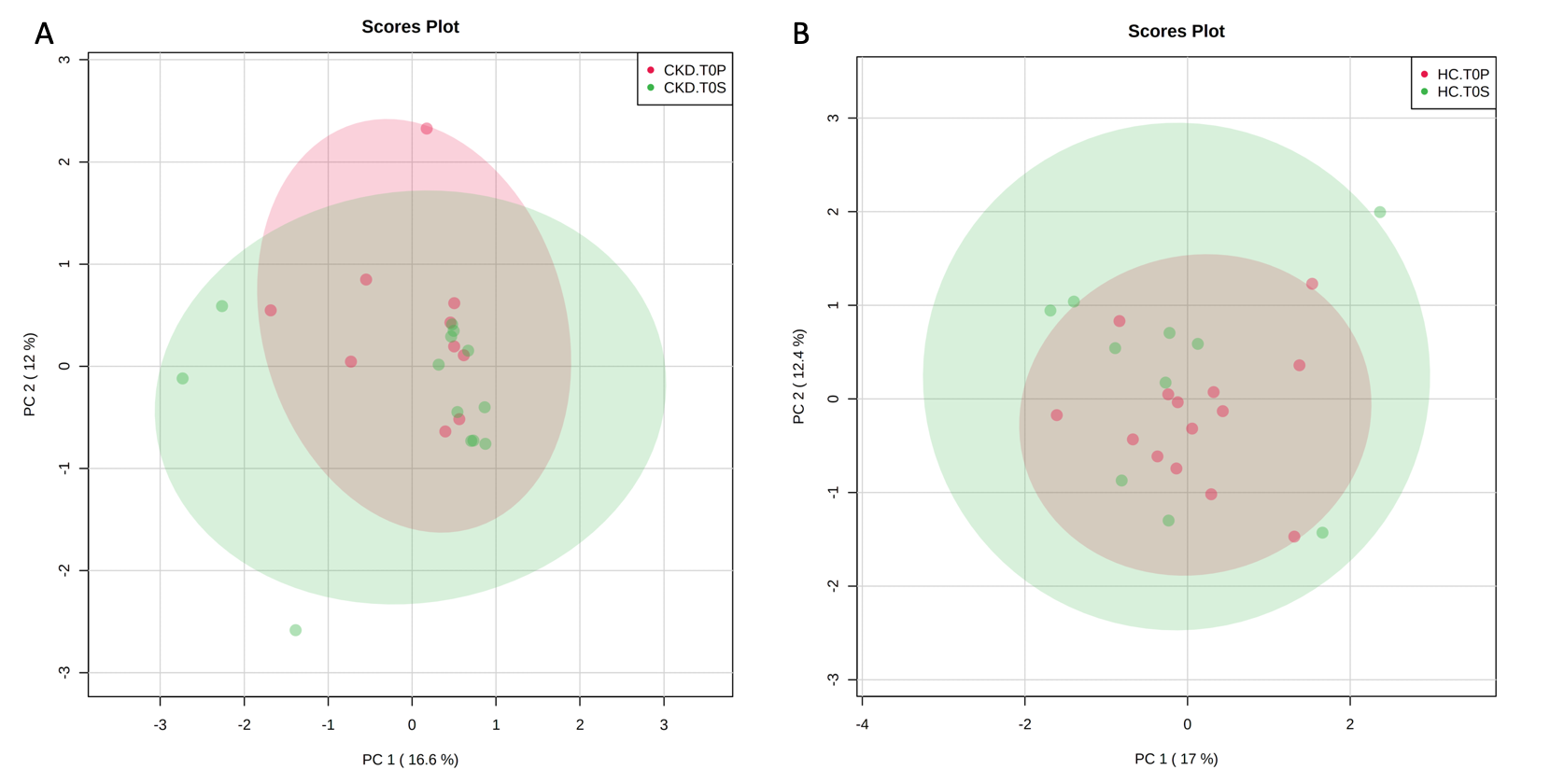


**Supplementary Figure S1.** Principal component analysis (PCA) of gut microbiota based on taxa with a relative abundance >0.1% and prevalence >20% within all samples found at baseline (T0) in CKD patients (panel A) and healthy controls (HC; panel B) after the randomization in placebo (P, red circles) or synbiotics (S, green circles) arms.


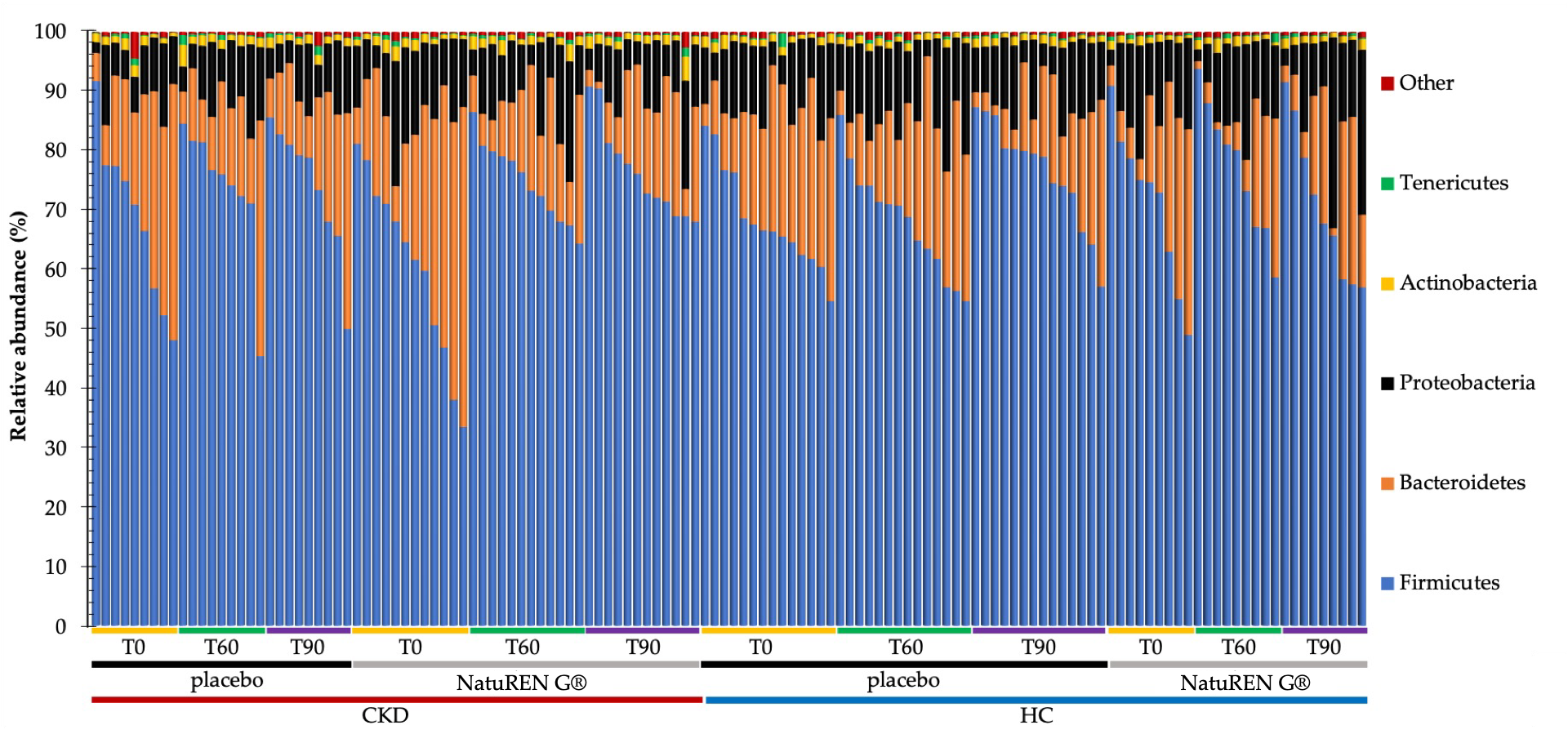


**Supplementary Figure S2.** Microbiota relative abundance at phylum level found in stool samples of chronic kidney disease (CKD) patients and healthy controls (HC), who took the placebo (P) or NatuREN G (S). Samples were delivered at placebo (P, red circles) or synbiotics (S, green circles) subgroups. Samples were delivered at 3 different times: run-in (T0), after 60 days of treatment (T60), and after 30 days of wash-out (T90).

# Supplementary Tables

**Supplementary Table S1.** Primers pairs employed for qPCR reactions and quantifications.

| **Bacterial genus** | **Target** | **Sequence (5’ - 3’)** | **bp** | **Annealing Temperature** | **References** |
| --- | --- | --- | --- | --- | --- |
| *Lactobacillus* genus | rRNA 16S | Lac-F: AGCAGTAGGGAATCTTCCA | 341 | 58 | Kwok et al., 2014 |
|  |  | Lac-R: CACCGCTACACATGGAG |  |  |  |
| *Bifidobacterium* genus | rRNA 16S | Bifid-F: CTCCTGGAAACGGGTGG | 550 | 55 | Kwok et al., 2014 |
|  |  | Bifid-R: GGTGTTCTTCCCGATATCTACA |  |  |  |

**Supplementary Table S2.** Dietary intake of the enrolled chronic kidney disease (CKD) patients and healthy controls (HC) allocated in placebo (P) or synbiotics (S) arm. Mean values and standard deviations (separated by “|”) refer to the last three-days before the sample delivery (baseline –T0, after 60 days of treatment –T60, and after further 30 days of wash out –T90).

|  | **CKD-S** | | | **CKD-P** | | | **HC-S** | | | **HC-P** | | |
| --- | --- | --- | --- | --- | --- | --- | --- | --- | --- | --- | --- | --- |
| **Dietary intake** | **T0**  (n.^$^ 13) | **T60**  (n. 13) | **T90**  (n. 13) | **T0**  (n. 10) | **T60**  (n. 9) | **T90**  (n. 10) | **T0**  (n. 10) | **T60**  (n. 10) | **T90**  (n. 10) | **T0**  (n. 14) | **T60**  (n. 14) | **T90**  (n. 14) |
| Energy kcal/day | 1814.7\|415.2 | 1703.9\|518.0 | 1795.1\|579.4 | 1669.2\|346.1§ | 1780.4\|405.6 | 1748.3\|415.7 | 2159.1\|416.3 | 2088.6\|548.7 | 1960.9\|458.7 | 2025.1\|466.8 | 1820.4\|310.9#§ | 1881.8\|381.7 |
| Carbohydrates g/day | 232.9\|62.1 | 207.2\|74.8 | 229.7\|81.5 | 194.3\|58.2 | 234.4\|81.8 | 227.3\|74.4 | 270.0\|57.9 | 264.0\|76.0 | 258.2\|84.5 | 254.2\|79.7 | 222.5\|61.7 | 235.1\|61.6 |
| Fiber g/day | 19.1\|7.0 | 22.6\|6.8# | 17.0\|5.6 | 20.5\|8.3 | 19.9\|8.0 | 22.6\|9.1 | 18.6\|4.7 | 23.6\|4.4# | 20.6\|4.8 | 22.9\|5.6 | 19.7\|4.6# | 20.7\|5.5 |
| Soluble fiber g/day | 6.7\|3.1 | 10.8\|3.1# | 5.7\|2.2 | 7.7\|3.6 | 7.2\|3.6 | 8.2\|4.0 | 6.1\|1.9 | 11.2\|1.7# | 6.5\|1.0 | 6.9\|2.0 | 6.4\|1.6 | 6.5\|2.0 |
| Insoluble fiber g/day | 12.4\|4.4 | 11.8\|3.8 | 11.2\|3.5 | 12.8\|4.8 | 12.8\|4.5 | 14.4\|5.4 | 12.5\|3.5 | 12.5\|3.1 | 14.0\|4.0 | 16.0\|3.8 | 13.3\|3.3# | 14.2\|3.8# |
| Total proteins g/day | 67.9\|15.4 | 67.7\|17.3 | 73.9\|31.1 | 57.8\|9.6 | 63.0\|16.5 | 61.8\|14.4 | 88.2\|19.0 | 83.8\|24.7 | 84.0\|15.8 | 84.6\|20.3 | 78.2\|12.0 | 78.8\|16.1 |
| Animal proteins g/day | 35.6\|8.4 | 39.2\|10.3 | 42.2\|25.7 | 36.0\|3.9 | 34.8\|8.5 | 34.3\|7.4 | 49.2\|15.5 | 47.0\|19.3 | 45.3\|12.3 | 44.9\|14.5 | 43.8\|13.1 | 43.8\|12.4 |
| Vegetable proteins g/day | 32.4\|8.7 | 28.5\|9.2# | 31.7\|9.3 | 21.8\|7.3 | 28.1\|9.4 | 27.4\|9.6 | 39.0\|7.7 | 36.8\|9.1 | 38.8\|11.7 | 39.6\|10.9 | 34.4\|8.6 | 35.6\|8.5 |
| Ratio P/F | 3.8\|0.7 | 3.1\|0.6# | 5.0\|3.7 | 3.3\|1.5 | 3.6\|1.4 | 3.1\|1.2 | 5.1\|2.0 | 3.6\|1.0# | 4.3\|1.2 | 3.8\|0.8 | 4.1\|1.0 | 4.0\|0.9 |
| Saturated FA g/day | 20.2\|5.6 | 21.0\|6.4 | 20.3\|7.8 | 20.4\|3.7 | 21.7\|4.8 | 20.3\|5.0 | 30.4\|8.9 | 29.9\|12.8 | 26.0\|8.0# | 23.9\|5.3 | 21.9\|3.5 | 22.1\|5.0 |
| PUFA g/day | 9.2\|1.9 | 9.5\|3.3 | 10.3\|4.8 | 8.8\|3.3 | 9.6\|2.6 | 8.9\|3.2 | 13.2\|3.7 | 13.0\|4.6 | 10.3\|1.9 | 16.7\|6.5 | 13.8\|6.6# | 13.3\|4.6# |
| Cholesterol mg/day | 132.8\|36.7 | 158.0\|62.6 | 165.5\|131.6 | 169.4\|95.4 | 138.6\|54.5 | 131.0\|43.7 | 231.7\|55.7 | 241.8\|101.7 | 204.0\|52.3 | 200.1\|65.6 | 176.0\|46.4 | 201.6\|59.3 |
| Na mg/day | 1347.6\|474.9 | 1242.2\|595.8 | 1293.8\|700.2 | 797.2\|282.5§ | 1207.2\|417.6# | 1109.8\|457.0#§ | 1499.8\|526.2 | 1571.3\|757.9 | 1411.4\|537.1 | 1525.7\|653.3 | 1342.3\|453.5§ | 1449.0\|568.6 |
| K mg/day | 2699.0\|729.3 | 2616.9\|663.1 | 2861.0\|852.1 | 2463.1\|511.1§ | 2542.5\|518.4 | 2731.6\|524.1 | 2847.1\|477.8 | 2891.4\|423.9 | 3167.5\|566.1 | 3443.9\|600.9§ | 3093.5\|443.4#§ | 3261.2\|659.5 |
| Cu mg/day | 9.4\|2.6 | 8.8\|2.3 | 9.6\|2.6 | 7.7\|1.9 | 8.2\|1.6 | 8.5\|1.7 | 10.1\|1.8 | 9.9\|1.8 | 10.8\|2.3 | 11.5\|2.3 | 10.1\|1.8# | 10.8\|2.4 |
| Ca mg/day | 574.7\|223.8 | 579.8\|209.1 | 537.1\|202.5 | 526.4\|189.5 | 506.9\|172.7 | 543.9\|146.6 | 727.6\|404.4 | 713.2\|551.7 | 715.4\|301.9 | 714.9\|213.6 | 685.6\|178.6 | 634.6\|145.6 |
| P mg/day | 940.5\|254.2 | 939.2\|260.7 | 987.1\|349.3 | 845.3\|119.4 | 876.7\|166.6 | 874.1\|160.1 | 1186.2\|330.6 | 1131.4\|428.5 | 1144.8\|258.9 | 1187.8\|276.2 | 1107.3\|125.5 | 1088.8\|173.5 |
| Vit B1 mg/day | 0.6\|0.2 | 0.6\|0.2 | 0.7\|0.2 | 0.5\|0.1 | 0.6\|0.1 | 0.6\|0.2 | 0.8\|0.2 | 0.8\|0.2 | 0.8\|0.2 | 1.0\|0.2 | 0.8\|0.2# | 0.9\|0.2 |
| Vit B2 mg/day | 1.2\|0.4 | 1.2\|0.4 | 1.3\|0.4 | 1.1\|0.3 | 1.1\|0.2 | 1.2\|0.3 | 1.4\|0.4 | 1.4\|0.3 | 1.4\|0.3 | 1.6\|0.4 | 1.4\|0.3 | 1.5\|0.2 |
| Vit B3 mg/day | 11.9\|1.9 | 12.2\|2.8 | 14.1\|5.7 | 11.0\|2.1 | 11.0\|2.4 | 11.4\|2.3 | 14.4\|3.8 | 13.4\|1.8 | 14.6\|1.8 | 14.6\|3.0 | 13.8\|3.0 | 14.3\|4.6 |
| Vit A µg/day | 743.3\|305.5 | 810.0\|310.7 | 829.9\|349.9 | 806.0\|273.5 | 727.2\|217.8 | 831.1\|268.7 | 844.6\|345.3 | 843.3\|282.4 | 947.2\|298.7 | 1011.4\|275.7§ | 902.3\|316.2 | 992.2\|256.0 |
| Vit C mg/day | 32.1\|11.8 | 32.2\|10.7 | 33.8\|15.0 | 31.7\|8.8 | 32.4\|10.4 | 36.5\|9.1 | 32.5\|11.8 | 34.6\|7.9 | 39.5\|13.0 | 44.4\|10.0 | 38.5\|9.4# | 42.4\|10.9 |
| Vit E mg/day | 5.6\|2.0 | 5.6\|1.9 | 5.9\|2.4 | 5.5\|1.7 | 5.8\|2.2 | 6.4\|1.8# | 5.8\|2.1 | 6.4\|1.6 | 7.0\|2.2 | 7.9\|1.9 | 7.0\|1.6# | 7.3\|2.1 |
| Vit B6 mg/day | 1.1\|0.2 | 1.1\|0.3 | 1.2\|0.5 | 0.9\|0.2 | 1.0\|0.2 | 1.0\|0.2 | 1.3\|0.3 | 1.2\|0.2 | 1.3\|0.2 | 1.5\|0.3 | 1.3\|0.3 | 1.4\|0.4 |
| Vit D µg/day | 1.4\|0.2 | 1.6\|0.3 | 1.6\|0.7 | 1.7\|0.6 | 1.5\|0.3 | 1.5\|0.3 | 1.7\|0.3 | 1.8\|0.4 | 1.7\|0.3 | 1.7\|0.3 | 1.7\|0.2 | 1.7\|0.4 |
| Folic acid µg/day | 188.0\|83.8 | 152.7\|117.8 | 167.8\|102.9 | 61.2\|47.8§ | 130.0\|91.2 | 110.5\|95.4# | 228.1\|100.8 | 200.5\|109.5 | 194.3\|88.8 | 198.0\|110.8 | 178.0\|89.6 | 181.8\|97.5 |
| Alcohol g/day | 9.5\|11.9 | 5.5\|9.9 | 7.5\|11.1 | 3.4\|6.6 | 2.0\|5.3 | 2.7\|5.3 | 1.4\|2.9 | 0.6\|1.3 | 2.4\|5.1 | 4.4\|5.7 | 3.2\|4.6 | 5.3\|6.5 |
| Eggs g/day | 5.2\|5.6 | 9.3\|10.8 | 8.2\|13.6 | 14.3\|25.0 | 4.4\|7.1 | 3.9\|6.8 | 14.6\|8.0 | 20.1\|14.3 | 12.4\|11.8 | 16.1\|11.6 | 11.7\|11.0 | 17.9\|16.8 |
| Fruits g/day | 295.0\|152.3 | 268.1\|143.7 | 296.2\|183.1 | 295.0\|146.2 | 311.1\|185.0 | 345.0\|173.9 | 277.9\|180.4 | 320.0\|137.8 | 360.0\|156.0 | 360.7\|121.2 | 353.6\|123.2 | 355.4\|157.6 |
| Legumes g/day | 30.7\|16.4 | 27.8\|10.4 | 19.7\|12.9# | 25.1\|12.1 | 28.4\|13.0 | 31.0\|19.4 | 32.0\|20.1 | 29.0\|28.0 | 38.0\|31.9 | 44.9\|22.0 | 32.2\|19.4 | 34.6\|21.8 |
| Meat g/day | 42.7\|8.8 | 50.2\|22.2 | 62.6\|57.0 | 40.1\|13.7 | 37.0\|22.5 | 32.0\|15.9 | 73.6\|64.6 | 56.7\|23.7 | 58.4\|17.3 | 63.3\|39.7 | 59.3\|46.9 | 61.1\|41.3 |
| Milk &der g/day | 111.0\|103.4 | 120.9\|95.8 | 117.4\|97.6 | 143.9\|100.6 | 129.8\|114.3 | 108.0\|100.6 | 147.5\|78.7 | 111.6\|96.9# | 121.6\|77.6 | 182.6\|133.5 | 154.1\|104.6 | 140.6\|106.6 |
| Preserved meat g/day | 14.6\|11.6 | 8.8\|8.9 | 12.6\|10.5 | 10.1\|10.8 | 9.9\|8.7 | 12.8\|12.5 | 11.7\|9.6 | 15.1\|15.9 | 15.5\|15.5 | 11.6\|14.4 | 15.0\|15.2 | 17.7\|14.9 |
| Ripened cheeses g/day | 17.8\|10.7 | 17.4\|13.1 | 12.7\|11.5# | 14.5\|11.2 | 12.7\|6.2 | 16.6\|10.1 | 24.1\|27.5 | 26.9\|39.9 | 23.9\|25.8 | 13.3\|12.7 | 16.8\|18.0 | 10.1\|11.8 |
| Sweets g/day | 41.8\|25.5 | 43.5\|24.1 | 52.7\|42.9 | 47.5\|30.2 | 57.5\|46.6 | 57.3\|44.8 | 75.8\|27.2 | 80.1\|44.7 | 70.3\|43.6 | 47.1\|33.9 | 35.1\|27.1 | 45.9\|33.8 |
| Tea g/day | 39.6\|74.3 | 41.8\|73.4 | 15.4\|55.5 | 75.0\|97.9 | 70.6\|90.1 | 48.6\|84.2 | 20.0\|63.2 | 40.0\|84.3 | 45.0\|95.6 | 120.4\|147.9 | 83.7\|123.0 | 120.4\|167.4 |
| Vegetable milk &der g/day | 5.8\|20.8 | 0.0\|0.0 | 0.0\|0.0 | 0.0\|0.0 | 0.0\|0.0 | 0.0\|0.0 | 0.0\|0.0 | 0.0\|0.0 | 0.0\|0.0 | 14.3\|53.5 | 28.6\|72.6 | 28.6\|72.6 |
| Vegetables g/day | 226.9\|118.3 | 247.3\|117.4 | 265.4\|142.0 | 240.0\|93.7 | 211.1\|96.1 | 260.0\|115.0 | 233.6\|133.0 | 230.0\|122.9 | 285.0\|133.4 | 317.9\|106.7 | 276.5\|130.8 | 317.9\|110.3 |
| Carbohydrates % kcal | 47.9\|4.8 | 44.9\|4.4 | 47.3\|4.5 | 42.9\|6.6 | 48.1\|8.0 | 47.8\|8.0# | 46.9\|5.6 | 47.1\|4.8 | 48.8\|6.0 | 46.3\|4.8 | 45.0\|5.8 | 46.6\|5.0 |
| Proteins % kcal | 15.1\|1.9 | 16.3\|2.8 | 16.6\|3.3 | 14.2\|3.0 | 14.4\|2.7 | 14.4\|2.2 | 16.4\|2.3 | 16.1\|1.9 | 17.4\|1.8 | 16.9\|2.6 | 17.5\|3.6 | 17.0\|2.7 |
| PUFA % kcal | 4.6\|0.6 | 5.0\|0.8 | 5.1\|1.0 | 4.8\|1.2 | 5.0\|1.1 | 4.7\|1.3 | 5.6\|1.5 | 5.6\|1.5 | 4.8\|0.9 | 7.4\|2.4 | 6.7\|2.4 | 6.4\|1.8 |
| Saturated FA % kcal | 9.9\|1.1 | 11.3\|1.6# | 10.2\|2.3 | 11.2\|1.6 | 11.2\|1.8 | 10.6\|2.0 | 12.6\|1.6 | 12.6\|2.3 | 11.8\|1.5 | 10.8\|2.2 | 11.0\|2.0 | 10.8\|2.0 |

* Expressed as tocopherol equivalent; $ n. indicates the number of filled questionnaires. #p-value <0.05 (ANOVA test within the same subgroup). §Comparing the same time (T0, T60 or T90), p-value <0.05 (Student’s t-test, P vs S). **Abbreviations**: P/F, ratio protein/fiber; FA, fatty acids; PUFA, polyunsaturated FA; milk &der, milk and derivates.

**Supplementary Table S3.** Means (|SD) of the identified species’ number (OTU) and Shannon diversity index of chronic kidney disease (CKD) patients and healthy controls (HCs) fecal microbiota based on allocation into the placebo (P) or synbiotics arm (S). Samples were collected at three times: run-in (T0), after 60 days of treatment (T60), and after 30 days of wash-out (T90).

| **Cohort** | **Randomization** | **Time point** | **ASVs** | **Shannon’s Index** |
| --- | --- | --- | --- | --- |
| **CKD** | **S** | **T0** | 290.92\|54.67 ^a^ | 2.31\|0.14 ^a^ |
|  |  | **T60** | 331.33\|52.17 ^b^ | 2.26\|0.28 ^a^ |
|  |  | **T90** | 338.67\|53.78 ^b^ | 2.20\|0.12 ^a^ |
|  | **P** | **T0** | 292.25\|65.85 ^a^ | 2.28\|0.21 ^a^ |
|  |  | **T60** | 340.75\|63.23 ^ab^ | 2.28\|0.25 ^a^ |
|  |  | **T90** | 347.00\|53.54 ^b^ | 2.34\|0.20 ^a^ |
| **HC** | **S** | **T0** | 276.67\|51.41 ^a^ | 2.20\|0.19 ^a^ |
|  |  | **T60** | 288.89\|53.46 ^b^ | 2.19\|0.22 ^a,#^ |
|  |  | **T90** | 288.56\|88.19 ^ab^ | 2.25\|0.22 ^a^ |
|  | **P** | **T0** | 284.21\|39.13 ^a^ | 2.30\|0.20 ^a^ |
|  |  | **T60** | 303.57\|40.80 ^b^ | 2.39\|0.21 ^b,#^ |
|  |  | **T90** | 289.00\|55.75 ^ab^ | 2.30\|0.17 ^a^ |

^a-b^ different superscript letters within the same subgroup (CKD-S, CKD-P, HC-S, and HC-P) showed a significant difference (*pval* <0.05; one way ANOVA test).
# p-value <0.05 between values found in CKD-S and CKD-P profiled at the same time point (T60).

**Supplementary Table S4.** The MaAsLin2 regression model showed the significant differences (*P* <0.05 and qval <0.5) in gut microbiota of chronic kidney disease (CKD) patients and healthy controls (HCs) treated with the innovative synbiotic (S) or plabebo (P) for 60 days (T60) and following 30 days of wash out (T90). Differences are showed comparing the follow-ups (T60 and T90) to the relative baseline (T0). Table header includes the coefficent of regression (coef), the standard error (stderr), total number of fitted cases (N), number of non-zero notations for the variable included within the same row (N.not.0), p-value (pval), and adjusted pval (qval).

| **Taxon** | **Group** | **Time point** | **coef** | **stderr** | **N** | **N.not.0** | **pval** | **qval** |
| --- | --- | --- | --- | --- | --- | --- | --- | --- |
| *Bacteroidetes* | **CKD-S** | * T60 | -0.957 | 0.423 | 38 | 38 | 0.030 | 0.371 |
| *Bacteroidetes* | **CKD-S** | * T90 | -1.187 | 0.415 | 38 | 38 | 0.007 | 0.247 |
| *Firmicutes* | **CKD-S** | * T60 | 0.343 | 0.103 | 38 | 38 | 0.002 | 0.102 |
| *Firmicutes* | **CKD-S** | * T90 | 0.373 | 0.101 | 38 | 38 | 0.001 | 0.079 |
| *Coriobacteriaceae* | **CKD-S** | * T60 | 0.896 | 0.440 | 38 | 38 | 0.049 | 0.483 |
| *Coriobacteriaceae* | **CKD-S** | * T90 | 0.886 | 0.431 | 38 | 38 | 0.047 | 0.483 |
| *Flavobacteriaceae* | **CKD-S** | * T60 | -1.267 | 0.528 | 38 | 38 | 0.022 | 0.360 |
| *Flavobacteriaceae* | **CKD-S** | * T90 | -1.691 | 0.518 | 38 | 38 | 0.002 | 0.102 |
| *Blautia* | **CKD-S** | * T60 | 0.617 | 0.262 | 38 | 38 | 0.024 | 0.360 |
| *Blautia* | **CKD-S** | * T90 | 1.020 | 0.257 | 38 | 38 | 0.000 | 0.072 |
| *Actinomycetaceae* | **CKD-S** | T60 | 1.246 | 0.575 | 38 | 37 | 0.037 | 0.410 |
| *Alcaligenaceae* | **CKD-S** | T90 | -2.394 | 0.905 | 38 | 38 | 0.012 | 0.319 |
| *Bacteroidaceae* | **CKD-S** | T90 | -1.141 | 0.490 | 38 | 38 | 0.026 | 0.360 |
| *Desulfovibrionaceae* | **CKD-S** | T90 | -1.208 | 0.514 | 38 | 38 | 0.024 | 0.360 |
| *Lachnospiraceae* | **CKD-S** | T90 | 0.745 | 0.227 | 38 | 38 | 0.002 | 0.102 |
| *Odoribacteraceae* | **CKD-S** | T90 | -1.829 | 0.799 | 38 | 35 | 0.028 | 0.370 |
| *Sphingobacteriaceae* | **CKD-S** | T90 | -2.025 | 0.780 | 38 | 37 | 0.014 | 0.319 |
| *Bacteroides* | **CKD-S** | T90 | -1.140 | 0.487 | 38 | 38 | 0.025 | 0.360 |
| *Pedobacter* | **CKD-S** | T90 | -2.037 | 0.857 | 38 | 37 | 0.023 | 0.360 |
| *Ruminococcus* | **CKD-S** | T90 | 0.623 | 0.280 | 38 | 38 | 0.033 | 0.382 |
| *Sutterella* | **CKD-S** | T90 | -2.683 | 1.004 | 38 | 34 | 0.011 | 0.319 |
| *Lachnospira* | **HC-P** | * T60 | -0.809 | 0.269 | 42 | 42 | 0.005 | 0.143 |
| *Lachnospira* | **HC-P** | * T90 | -0.593 | 0.269 | 42 | 42 | 0.034 | 0.414 |
| *Bacteroidetes* | **HC-P** | T90 | -0.950 | 0.395 | 42 | 42 | 0.021 | 0.334 |
| *Firmicutes* | **HC-P** | T90 | 0.166 | 0.070 | 42 | 42 | 0.023 | 0.334 |
| *Bacteroidaceae* | **HC-P** | T90 | -0.998 | 0.396 | 42 | 42 | 0.016 | 0.334 |
| *Enterobacteriaceae* | **HC-P** | T60 | 2.882 | 0.843 | 42 | 42 | 0.001 | 0.143 |
| *Flavobacteriaceae* | **HC-P** | T90 | -1.022 | 0.431 | 42 | 42 | 0.023 | 0.334 |
| *Odoribacteraceae* | **HC-P** | T90 | -2.445 | 0.677 | 42 | 38 | 0.001 | 0.143 |
| *Porphyromonadaceae* | **HC-P** | T90 | -1.442 | 0.662 | 42 | 42 | 0.035 | 0.414 |
| *Bacteroides* | **HC-P** | T90 | -0.993 | 0.393 | 42 | 42 | 0.016 | 0.334 |
| *Blautia* | **HC-P** | T90 | 0.523 | 0.222 | 42 | 42 | 0.024 | 0.334 |
| *Butyricimonas* | **HC-P** | T90 | -2.068 | 0.792 | 42 | 30 | 0.013 | 0.334 |
| *Coprococcus* | **HC-P** | T60 | -0.796 | 0.263 | 42 | 42 | 0.004 | 0.143 |
| *Dorea* | **HC-P** | T90 | 0.672 | 0.320 | 42 | 42 | 0.042 | 0.445 |
| *Enterobacter* | **HC-P** | T60 | 2.703 | 0.852 | 42 | 41 | 0.003 | 0.143 |
| *Escherichia* | **HC-P** | T60 | 2.302 | 0.928 | 42 | 35 | 0.018 | 0.334 |
| *Flavobacterium* | **HC-P** | T90 | -1.494 | 0.707 | 42 | 40 | 0.041 | 0.445 |
| *Parabacteroides* | **HC-P** | T90 | -1.575 | 0.703 | 42 | 42 | 0.031 | 0.405 |
| *Serratia* | **HC-P** | T60 | 2.860 | 0.868 | 42 | 42 | 0.002 | 0.143 |
| *Trabulsiella* | **HC-P** | T60 | 2.632 | 0.879 | 42 | 35 | 0.005 | 0.143 |

*AVSs reaching significance (P <0.05 and qval <0.5) at both follow-ups (T60 and T90) compared against the related baseline (T0).

**Supplementary Table S5.** Fecal volatile organic compounds (VOCs) significantly different (μg/g) between baseline (T0) and 60 days of treatment (T60) after placebo (P) or the synbiotic (S) administration in chronic kidney disease (CKD) patients and healthy controls (HCs).

| **Metabolites** | **Group** | **Treatment** | **T0** | **T60** | **p-value** |
| --- | --- | --- | --- | --- | --- |
| Acetic Acid | HC | P | 3.42 | 1.96 | ^(*)^ ns |
|  |  | S | 2.46 | 1.94 | ns |
|  | CKD | P | 1.53 | 2.09 | ns |
|  |  | S | 1.72 | 2.53 | 0.048 |
| Propanoic Acid | HC | P | 1.58 | 1.03 | ns |
|  |  | S | 1.19 | 0.83 | ns |
|  | CKD | P | 0.76 | 1.29 | ns |
|  |  | S | 1.08 | 1.53 | 0.028 |
| *iso-*butyric acid | HC | P | 0.37 | 0.17 | ns |
|  |  | S | 0.22 | 0.10 | 0.041 |
|  | CKD | P | 0.02 | 0.01 | ns |
|  |  | S | 0.23 | 0.34 | ns |
| 3-Methylvaleric acid | HC | P | 1.80 | 1.02 | ns |
|  |  | S | 1.61 | 0.60 | 0.001 |
|  | CKD | P | 0.99 | 1.52 | ns |
|  |  | S | 1.28 | 1.67 | ns |
| Nonanoic acid | HC | P | 2.62 | 2.71 | ns |
|  |  | S | 3.12 | 2.37 | ns |
|  | CKD | P | 1.79 | 1.87 | ns |
|  |  | S | 3.15 | 2.81 | 0.021 |
| Ethyl butyrate | HC | P | 3.73 | 2.06 | ns |
|  |  | S | 1.30 | 0.30 | 0.048 |
|  | CKD | P | 3.62 | 2.20 | ns |
|  |  | S | 0.40 | 0.16 | ns |
| Ethyl valerate | HC | P | 3.16 | 0.61 | ns |
|  |  | S | 1.43 | 0.37 | 0.034 |
|  | CKD | P | 2.28 | 1.08 | ns |
|  |  | S | 0.49 | 0.44 | ns |
| Ethyl 4-methylpentanoate | HC | P | 0.23 | 0.04 | ns |
|  |  | S | 0.12 | 0.00 | 0.049 |
|  | CKD | P | 0.17 | 0.18 | ns |
|  |  | S | 0.03 | 0.00 | ns |
| Butyl butyrate | HC | P | 2.51 | 0.91 | ns |
|  |  | S | 0.58 | 0.26 | 0.009 |
|  | CKD | P | 0.56 | 0.79 | ns |
|  |  | S | 0.63 | 1.26 | ns |
| Propyl valerate | HC | P | 1.14 | 0.66 | ns |
|  |  | S | 0.53 | 0.11 | 0.012 |
|  | CKD | P | 0.24 | 0.67 | ns |
|  |  | S | 0.59 | 0.45 | ns |
| Butyl valerate | HC | P | 1.11 | 0.25 | ns |
|  |  | S | 0.63 | 0.15 | 0.035 |
|  | CKD | P | 0.49 | 0.76 | ns |
|  |  | S | 0.45 | 0.73 | ns |
| 2-Carbomethoxyphenol | HC | P | 0.37 | 0.30 | ns |
|  |  | S | 0.19 | 0.25 | ns |
|  | CKD | P | 0.18 | 0.24 | 0.029 |
|  |  | S | 0.27 | 0.35 | ns |
| Ethyl pentadecanoate | HC | P | 0.00 | 0.05 | ns |
|  |  | S | 0.08 | 0.02 | 0.036 |
|  | CKD | P | 0.14 | 0.13 | ns |
|  |  | S | 0.04 | 0.07 | ns |
| 1-Pentanol | HC | P | 0.24 | 0.11 | ns |
|  |  | S | 0.23 | 0.08 | 0.021 |
|  | CKD | P | 0.09 | 0.15 | ns |
|  |  | S | 0.02 | 0.07 | ns |
| 1-Hexadecanol | HC | P | 0.66 | 0.60 | ns |
|  |  | S | 0.52 | 0.41 | ns |
|  | CKD | P | 0.26 ^b^ | 0.32 | ns |
|  |  | S | 0.93 ^a^ | 0.36 | ns |
| 2-Undecanone | HC | P | 0.10 | 0.18 | 0.036 |
|  |  | S | 0.13 | 0.08 | ns |
|  | CKD | P | 0.08 ^b^ | 0.77 | ns |
|  |  | S | 0.13 ^a^ | 0.24 | ns |
| 2-Tridecanone | HC | P | 0.02 | 0.06 | 0.023 |
|  |  | S | 0.05 | 0.07 | ns |
|  | CKD | P | 0.03 | 0.07 | ns |
|  |  | S | 0.06 | 0.10 | 0.048 |
| 2-Nonadecanone | HC | P | 0.00 | 0.00 | ns |
|  |  | S | 0.08 | 0.03 | 0.046 |
|  | CKD | P | 0.01 | 0.01 | ns |
|  |  | S | 0.03 | 0.07 | ns |
| Octanal | HC | P | 0.02 | 0.09 | ns |
|  |  | S | 0.06 | 0.11 | ns |
|  | CKD | P | 0.01 ^b^ | 0.06 | ns |
|  |  | S | 0.12 ^a^ | 0.11 | ns |
| Carbon disulfide | HC | P | 3.11 | 3.89 | ns |
|  |  | S | 2.20 | 2.92 | ns |
|  | CKD | P | 2.74 | 4.45 | 0.027 |
|  |  | S | 4.69 | 4.77 | ns |
| Dimethyl trisulfide | HC | P | 0.93 | 0.08 | ns |
|  |  | S | 0.23 | 0.14 | ns |
|  | CKD | P | 0.12 | 0.04 | ns |
|  |  | S | 0.24 | 0.05 | 0.044 |
| Decane | HC | P | 0.29 | 0.08 | ns |
|  |  | S | 0.18 | 0.40 | ns |
|  | CKD | P | 0.20 | 0.67 | ns |
|  |  | S | 0.25 | 0.84 | 0.041 |
| Heneicosane | HC | P | 0.06 | 0.19 | 0.023 |
|  |  | S | 0.20 | 0.05 | 0.036 |
|  | CKD | P | 0.35 | 0.25 | ns |
|  |  | S | 0.23 | 0.25 | ns |
| γ-Terpinene | HC | P | 0.57 | 0.16 | ns |
|  |  | S | 0.07 | 0.16 | ns |
|  | CKD | P | 0.25 | 1.05 | 0.035 |
|  |  | S | 0.02 | 0.14 | ns |
| 3-Carene | HC | P | 0.39 | 0.63 | ns |
|  |  | S | 0.24 | 2.03 | ns |
|  | CKD | P | 0.15 | 0.04 ^b^ | ns |
|  |  | S | 1.39 | 0.25 ^a^ | ns |
| 3-Ethyltoluene | HC | P | 0.26 | 0.32 | ns |
|  |  | S | 0.03 | 0.18 | ns |
|  | CKD | P | 0.14 | 0.35 | 0.016 |
|  |  | S | 0.52 | 0.68 | ns |
| Estragole | HC | P | 0.15 | 0.05 | ns |
|  |  | S | 0.13 | 0.08 | 0.026 |
|  | CKD | P | 0.91 | 0.09 | ns |
|  |  | S | 0.04 | 0.34 | ns |
| Ethyl phenylacetate | HC | P | 0.15 | 0.04 | 0.032 |
|  |  | S | 0.07 | 0.03 | 0.037 |
|  | CKD | P | 0.23 | 0.05 | ns |
|  |  | S | 0.00 | 0.00 | ns |
| O-Cymene | HC | P | 2.13 | 0.49 ^a^ | ns |
|  |  | S | 0.17 | 0.38 ^b^ | ns |
|  | CKD | P | 0.20 | 0.52 | ns |
|  |  | S | 0.15 | 0.09 | ns |
| Durene | HC | P | 0.36 ^a^ | 0.40 | ns |
|  |  | S | 0.12 ^b^ | 0.17 | ns |
|  | CKD | P | 0.11 | 0.36 | ns |
|  |  | S | 0.29 | 0.65 | ns |

(*) ns: not significant (Student’s t-test).

(a-b) At the same time and within HC or CKD groups, values of each compound have different superscript letters when are significantly different (*P* <0.05; Student’s t-test).
